# *Brugia malayi* and other filarial parasite species in animals in areas endemic for lymphatic filariasis in Belitung District, Indonesia

**DOI:** 10.1371/journal.pntd.0013593

**Published:** 2025-10-07

**Authors:** Irina Diekmann, Taniawati Supali, Kerstin Fischer, Elisa Iskandar, Noviani Sugianto, Yossi Destani, Rahmat Alfian, Gary J. Weil, Peter U. Fischer

**Affiliations:** 1 Infectious Diseases Division, Department of Medicine, Washington University School of Medicine, St. Louis, Missouri, United States of America; 2 Department of Parasitology, Faculty of Medicine, Universitas Indonesia, Jakarta, Indonesia; Xuzhou Medical University, CHINA

## Abstract

**Background:**

*Brugia malayi* is the most common cause of lymphatic filariasis (LF) in Indonesia. A zoophilic ecotype that infects both humans and animals occur in Belitung District in Indonesia. The district received five annual rounds of mass drug administration (MDA) between 2006 and 2010 and passed three transmission assessment surveys (TAS) in subsequent years. However, a survey in five villages in 2021 showed a microfilaria (Mf) prevalence of 2.1% in humans. The reappearance of *B. malayi* infection in humans may be due to reintroduction from animal reservoirs. The goal of this study was to determine *B. malayi* prevalence in potential reservoir hosts and to improve the identification of filarial Mf found in animals.

**Methodology/principal findings:**

Venous blood was collected from 291 cats, 41 dogs, and 163 crab-eating macaques (*Macaca fascicularis*) from areas with and without human *B. malayi* infection. *B. malayi* Mf were detected by microscopy in 1.4%, 7.3% and 13.5% of the samples, respectively. The geometric mean Mf density varied from 133 Mf/mL(dogs) to 255 Mf/mL (macaques). While *Brugia* Mf were easily differentiated from *Dirofilaria* Mf by microscopy, the morphological differentiation between *B. malayi* and *B. pahangi* was not reliable. qPCR detected *B. malayi* DNA in blood from 4.1% of cats, 2.4% dogs, and 13.5% macaques. In addition, infections or co-infection with *B. pahangi* (cats, dogs) or *D. immitis* (dogs) were detected. A novel *Dirofilaria* species was morphologically identified in 20.3% of macaques.

**Conclusions/significance:**

Microscopy was less accurate for detection and species identification of Mf than qPCR. The presence of *B. malayi* Mf in animals represents a challenge for the elimination of LF in some areas in Indonesia. More research is needed to better understand *B. malayi* transmission between animals and humans in endemic areas like Belitung where routine MDA may not be sufficient to eliminate LF.

## Author summary

*Brugia malayi* is the most common filarial parasite that causes lymphatic filariasis in Indonesia. It was thought to have been locally eliminated in Belitung district following five rounds of mass drug administration (MDA). However, post-MDA surveillance in 2021 identified persistent or recrudescent filarial infections in several villages with microfilaria prevalence as high as 5%. Possible explanations include undersampling of adults during routine surveillance or reintroduction of new infections from infected animals. The aim of this study was to determine the *B. malayi* infection rates in animals using blood samples from 291 cats, 41 dogs and 163 macaques and to compare Mf detection rates determined by microscopy and by qPCR. *B. malayi* was detected in 5.9% of the samples by microscopy and in 7.1% of the samples by qPCR. Apart from *B. malayi* three other filarial species were detected in the samples. It can be concluded that animals in Belitung are commonly infected with *B. malayi*, with macaques showing particularly high Mf prevalence and density compared to dogs and cats. This underlines the need for increased surveillance, targeted control measures, and further research to identify means of preventing zoonotic transmission of *B. malayi* from animals to humans.

## Introduction

Lymphatic filariasis (LF) is a mosquito-borne neglected tropical disease caused by the filarial parasites *Wuchereria bancrofti, Brugia malayi* and *B. timori*. The World Health Organization has targeted LF for elimination, with mass drug administration (MDA) of anti-filarial medications to all people at risk of infection as the key strategy. While the Global Program to Eliminate LF has made great progress in many countries, there are still 794 million people in 44 countries at risk of infection that require MDA [1]. About 90% of LF infections are caused by *W. bancrofti*, while the remaining 10% are caused by *Brugia* species, mostly in Southeast Asia [2,3]. Strains of these parasites differ in ecological requirements [4], vector species [5,6], periodicity of circulating microfilariae (Mf) [7,8] and definitive host species [9]. *W. bancrofti* and *B. timori* exclusively infect humans, whereas zoophilic strains of *B. malayi* infect also other mammals [9,10]. The infections in animals are usually asymptomatic; they rarely cause clinical symptoms such as lymphoedema or lymphadenopathy [2,11].

The majority of human LF in Indonesia is caused by *B. malayi*; nocturnally subperiodic zoophilic brugian filariasis is especially common in the western part of the country including Belitung Island and has been reported from humans, domestic and wild animals [7,12]. Indonesia’s national program to eliminate LF provided five annual rounds of MDA with diethycarbamazine plus albendazole with high coverage in Belitung between 2006 and 2010. Belitung district later passed three transmission assessment surveys (TAS) and was declared free of LF in 2017 by the Indonesian Ministry of Health. However, post-MDA surveys in 2021 documented Mf prevalences in adults in Belitung >1% in 5 out of 7 examined villages [13]. One explanation for the reappearance of *B. malayi* in humans could be mosquito transmission of parasites from an animal reservoir to the human population. More information was needed to understand whether zoonotic infections in Belitung were sufficient to sustain transmission from animals to humans.

Besides *B. malayi,* cats, dogs and macaques are natural hosts to a number of other filarial species*. B. pahangi* is mostly found in cats and dogs and rarely in humans. The morphological differentiation of Mf between this parasite and of *B. malayi* is challenging [14]. Several *Dirofilaria* species such as *D. immitis*, *D. repens* or *D. var ‘hongkongensis*’ are commonly found in cats, dogs and macaques, but these species have only been rarely reported to infect humans [15,16]. Macaques are hosts of *D. magnilarvatum*, *Wuchereria kalimantani*, *Ceropithifilaria* species and several other filarial species [17]. In rare circumstances these species can infect humans [18], but they have no public health relevance.

Other filarial species that infect potential reservoir hosts of *B. malayi* can pose a challenge for identification of the parasite by microcopy. The identification of Mf based on morphological features requires well-trained microscopists who can reliably identify the correct species. Microscopy results can be confirmed using PCR or qPCR [10,19–22]. Molecular assays together with DNA sequence information available in public databases should be useful for accurate identification of filarial species and strains.

In the present study, we have collected blood from cats, dogs and macaques in an area in Indonesia which is endemic for *B. malayi* in humans despite extensive efforts to eliminate the infection based on mass drug administration. We compared how morphological identification of Mf in animal blood aligned with molecular detection methods to determine the prevalence and density of filarial infections in animals. *B. malayi* was detected in all three animal species along with several other filarial species. However, the high prevalence and Mf density in macaques suggests that they are the most important enzootic reservoir for *B. malayi* in Belitung.

## Materials and methods

### Ethics approval and consent

The trapping and blood collection of animals was approved by the Ministries of Health and of Environment and Forestry of Indonesia (protocol# 22-040365). The study received an ethical approval from the ethical committee of Universitas Indonesia no 515/UN2.F1/ETIK/PPM.00.02/2022. Collection of blood from household pets was approved by the pet owners. Blood collection was performed by veterinary technicians under the supervision of veterinarians. Human samples were collected from adults during a surveillance study and was approved by the ethics committee of the Faculty of Medicine, Universitas Indonesia, Jakarta (number KET-650/UN2.F1/ETIK/PPM.00.02/2021). Informed oral consent according to local practices was obtained. Parasite samples were analyzed from deidentified study participants.

### Study area

Belitung island is administratively divided into two districts, Belitung and Belitung Timur. The study areas in Belitung district have been described in detail previously [13]. For the animal blood collection, villages with known presence of *B. malayi* microfilaremia in humans were selected. Blood samples were collected from cats and dogs in four endemic villages (Selat Nasik, Petaling, Kembiri, Lassar, Sulat Gual). Macaques (*Macaca fascicularis*) blood samples were obtained in three endemic villages (Selat Nasik, Petaling, Kembiri, Lassar) and in one control village without human infection (Kacang Butor) (S1 Table).

### Sample collection

**Fig 1:**
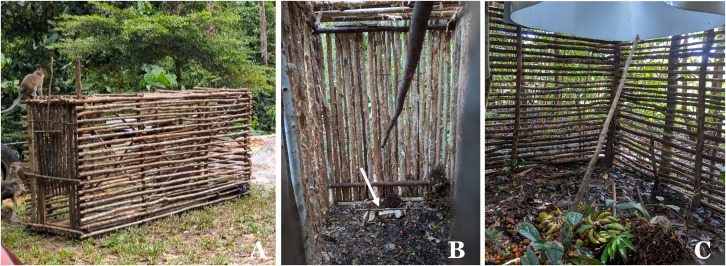
Cage traps for macaques: (A) Cage trap type A with manual falling door. (B) Cage trap type B with a trigger mechanism (arrow). (C) Cage trap type C with the entrance from the top.

Two different time points were chosen for blood sampling (between 07:30–10:30am or between 3:00–7:30pm). Data on the sex, community health center, village, sub-village, neighborhood association, and community association were recorded for every animal. Cats and dogs were differentiated between pets (associated with the name of the owner) and feral animals (which were not linked to a household) (S1 Table). Cats and dogs were caught using nets or directly restrained. Three different trap models were set up to catch macaques (Fig 1). Banana, cassava, jackfruit and palm oil fruits were offered as bait to attract the macaques. Cage trap type A was used in Kacang Butor where the animals lived in close proximity to humans, because the collection sites were near a tourist area where humans feed the macaques. The door was manually closed after the macaques entered the cage. The other traps were built to lure the macaques in areas where humans are normally not present. Cage trap Type B was built with a trigger mechanism. The fruits were placed behind a trigger that causes the entrance door to shut when animals are in the cage. Cage trap type C with an entrance from the top was the most successful trap; it allowed macaques to enter from the top to access fruit, but the animals were unable to escape due to a metal tube fixated at the entrance hole.

Additional data including GPS coordinates and habitat (forest near residence/ tourist attraction or palm oil plantation) were recorded for macaques (S1 Table). The macaques were sedated by intramuscular of ketamine HCL (10 mg/kg, Ket-A-100, Agrovet Market SA, Peru) in the hind limb. At the onset of the sedation the animals were caught with a fishing net to remove them from the cage trap. For each animal approximately 3 mL blood was obtained from the saphenous vein using the Becton Dickinson vacutainer system with EDTA tubes (Wayne, NJ, USA). A total of 495 blood samples were collected (291 cats, 41 dogs and 163 macaques) (S1 Table). None of the tested animals had any signs of filarial disease such as lymphedema. Animals and their respiratory rate were observed after blood collection and released after they were awake. Blood samples were transported at ambient temperature to the District Health Office, Belitung for Mf detection by microscopy. Plasma was separated from blood by centrifuging the blood samples for 5 min at 15,000 rpm within 24 hr. The plasma and the blood samples were frozen at -20°C. The blood samples were used for qPCR and further genomic analysis.

### Morphological identification and measurement of microfilariae

**Fig 2:**
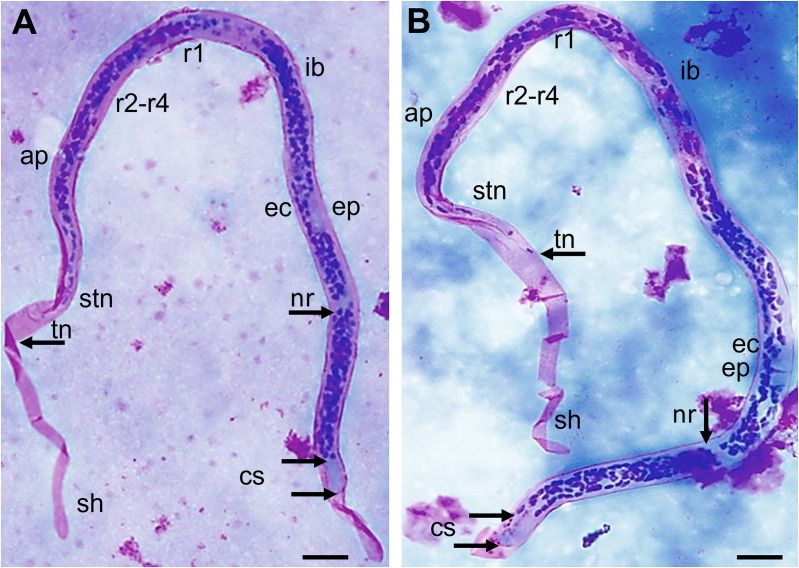
Morphological features of a Giemsa-stained *B. malayi* microfilaria from a macaque and a *B. pahangi* microfilaria from a dog. (A) *B. malayi* microfilaria from a macaque. (B) *B*. pahangi** microfilaria from a dog, cs- cephalic space; nr-nerve ring; ec-excretory cell; ep-excretory pore; ib-inner body or “Innenkörper”; ap-anal pore; r-1,r2-4-rectal cells; stn-subterminal nuclei; tn-terminal nucleus, arrows – measurement points, scale bar 10 μm.

The slides were examined by two experienced microscopists to identify and count Mf. Three-line thick blood smear (60 µl on the slide) was prepared as previously described [23]. Slides were air-dried for 2 days at ambient temperature and then stained with 3% Giemsa solution (Sigma-Aldrich, Germany). Species identification was based on features such as the present of a sheath, stained appearance of the sheath, measurement of total body length and width, cephalic space (length, width and their ratio), the location of nerve ring (NR), excretory pore (EP) and excretory cell (EC), anal pore (AP), genital pore (GP), the “Innenkörper“, and the amount and position of terminal nuclei (TN) in the tail structure of microfilariae [24] (Fig 2). For morphometric comparison, measurements were taken from 26 individual *B. malayi* and 28 *B. pahangi* Mf obtained from two different host species. Measurements of *B. malayi* Mf obtained from animals were compared to Mf obtained from deidentified human samples from the same study location [13] Measurements included length from head to tail (terminal nuclei), width measured at the NR level, length from head to nerve ring, length of the cephalic space clear of nuclei, and the ratio of the mid-body width divided by the length of the nuclei clear cephalic space (Fig 2). The measurements were taken, and photos were captured utilizing the microscope OLYMPUS BX40F4 (Olympus Optical Co. Ltd., Japan) with 1000x magnification using cellSens Standard 1.18 software (Olympus Corporation of the Americas, USA).

### DNA extraction and qPCR

Total DNA was extracted from 50 μL of animal blood (in EDTA) using Qiagen Blood and Tissue Kit (Hilden, Germany) according to the manufacturer’s protocol. The elution step contained 200 μL DEPC-treated water. The extracted DNA was kept in -20°C freezer until further use. For positive controls, 50 μL uninfected dog blood was mixed with one Mf of *B. malayi* or *B. pahangi* obtained from infected cat and dog blood provided by the NIH/NIAID Filariasis Research Reagent Resource Center (www.beiresources.org) or with one Mf obtained from a *D. immitis* positive dog field sample, confirmed through microscopy and qPCR, respectively. According to a previously described protocol [25] all *B. malayi* positive and selected samples with other species single Mf were picked for whole genome sequencing to confirm the species identification and for population genomic analysis. The whole genome results and analysis are not included in this manuscript and will be presented in a separate publication. Successful DNA extraction was confirmed by probe-based qPCR detecting the pan-filarial 28S region. All positive samples were further processed using species-specific qPCR that targeted the *Hha*I repeat for *B. malayi*, the mitochondrial cytochrome *c* oxidase subunit 1 (COI) for *D. immitis* and partial 12S with a new design probe for this study for *B. pahangi* [25–28]. PCR conditions and primer/probe sequences are provided in S4 Table.

### Statistical analyses

Descriptive statistics and morphometric comparisons using the Mann-Whitney test were performed using GraphPad 10.2.1. For Mf densities geometric means and medians were calculated. For morphometric comparisons the arithmetic mean was used because a standard distribution was assumed. The methodology reliability agreement of the morphological examination vs. that of qPCR was calculated using Cohen’s kappa κ coefficients with 95% confidence intervals (CIs) in the software package DescTools 0.99.45 in R [29]. The interpretation of the Cohen’s κ values based on the guidelines outlined by Landis and Koch [30].

## Results

### Morphological differentiation of microfilariae

The morphological differentiation between the sheathed *Brugia* and non-sheathed *Dirofilaria* Mf is straightforward. However, the morphological differentiation between *B. malayi* and *B. pahangi* can be challenging (Fig 2). The morphological and morphometric comparison of Mf from *B. malayi* obtained from a cat and macaques showed no differences, with exception of the length of the cephalic space clear of nuclei (Table 1). Apart from the length from the head to the nerve ring, the Mf obtained from humans were overall smaller compared to those from a cat and macaques, whereas the length of the cephalic space clear of nuclei was larger. The comparison between *B. pahangi* Mf obtained from a cat and dogs showed significant differences between length from head to tail and length from head to nerve ring, but the width and the length of the cephalic space clear of nuclei were similar. Measurements for Mf of *B. malayi* and *B. pahangi* were all significantly different except for the ratio width:nuclei free cephalic space. However, these morphometric differences between *B. malayi* and *B. pahangi* rely on statistical assessments. No single marker can be used to reliably speciate a single Mf.

**Table 1:** Morphometry of microfilariae of *B. malayi* and *B. pahangi* from different host species (in µm). Measurements include range and arithmetic mean ± SD.

	Host	Nr. of Mf measured	Length from head to tail	Width	Length from head to nerve ring	Length nuclei free cephalic space	Ratio-width: nuclei free cephalic space
*B. malayi*	Cat (n = 1)	5	196.6–223.6 | 209.9 ± 10.63	5.51–6.09 | 5.74 ± 0.25	36.07–43.96 | 40.88 ± 2.92	4.71–6.55 | 5.37 ± 0.73	0.79-1.17:1 | 0.94 ± 0.14
	Monkey (n = 3)	21	179.1–221 | 202.8 ± 10.27	4.14–9.64 | 6.472 ± 1.251	37.52–51.91 | 43.34 ± 4.089	5.12–9.18 | 6.50 ± 1.1	0.72-1.65:1 | 1.02 ± 0.25
	Cat/ monkey *p*-value		0.2	0.1098	0.2899	0.0147*	0.8158
	Human (n = 2)	20	168.6–216.0 | 191.8 ± 14.09	4.1–7.35 | 5.56 ± 0.77	35.16–51.7 | 41.66 ± 4.19	6.55–8.55 | 7.66 ± 0.67	0.89–1.81:1 | 1.4 ± 0.21
	Cat & monkey/ human p-value		0.0021**	0.0091**	0.2528	<0.0001**	<0.0001**
*B. pahangi*	Cat (n = 1)	13	222–262.3 | 246.4 ± 12.19	5.68–7.82 | 6.75 ± 0.66	43.34–73.84 | 53.32 ± 8.31	6.74–10.9 | 8.40 ± 1.49	0.98-1.61:1 | 1.25 ± 0.20
	Dog (n = 2)	15	185.3–255.6 | 222 ± 18.08	5.12–7.5 | 6.327 ± 0.761	40.76–52.3 | 45.51 ± 3.32	6.28–9.18 | 7.64 ± 0.81	0.87–1.76:1 | 1.24 ± 0.26
	*p*-value		0.0002***	0.1267	0.0040**	0.2745	0.9715
*B. malayi*	Cat & monkey & human	46	168.6–223.6 | 198.8 ± 13.54	4.1–9.64 | 5.99 ± 1.07	35.16–51.91 | 42.27 ± 4.05	4.71–9.18 | 6.9 ± 1.17	0.72–1.81:1 | 1.22 ± 0.3
*B. pahangi*	Cat & dog	28	185.3–262.3 | 233.4 ± 19.72	5.12–7.82 | 6.52 ± 0.74	40.76–73.84 | 49.27 ± 7.29	6.28–10.9 | 8.1 ± 1.22	0.87-1.76:1 | 1.25 ± 0.23
	*p*-value		<0.0001**	<0.0001**	0.0013**	0.0068**	0.7169

### Detection and species identification of microfilariae in animal blood by microscopy

Mf were detected in 16.2% (80/495) of the samples from animals. *B. malayi* was identified in 5.9% (29/495) of the animals (22 macaques, 4 cats and 3 dogs). The Mf prevalence of *B. malayi* was 1.4% in cats, 7.3% in dogs and 13.5% in macaques (S2 Table). Nine of these macaques (5.5%) were co-infected with *B. malayi* and an unknown *Dirofilaria* species. In total 34 (20.3%) blood samples from macaques contained Mf of *Dirofilaria* sp. Microfilariae in one sample obtained from a male macaque collected from the forest near a residence in the village Selat Nasik could not be clearly identified by morphology. *B. pahangi* was detected in 3.8% of the cats and in 7.3% of the dogs, but not in macaques. *D. immitis* was detected in 34.2% of the dogs, but not in cats or macaques. In one dog sample (2.4%) Mf of *D. immitis* and *B. malayi* were detected.

### Microfilaria density by host and filarial species

**Fig 3:**
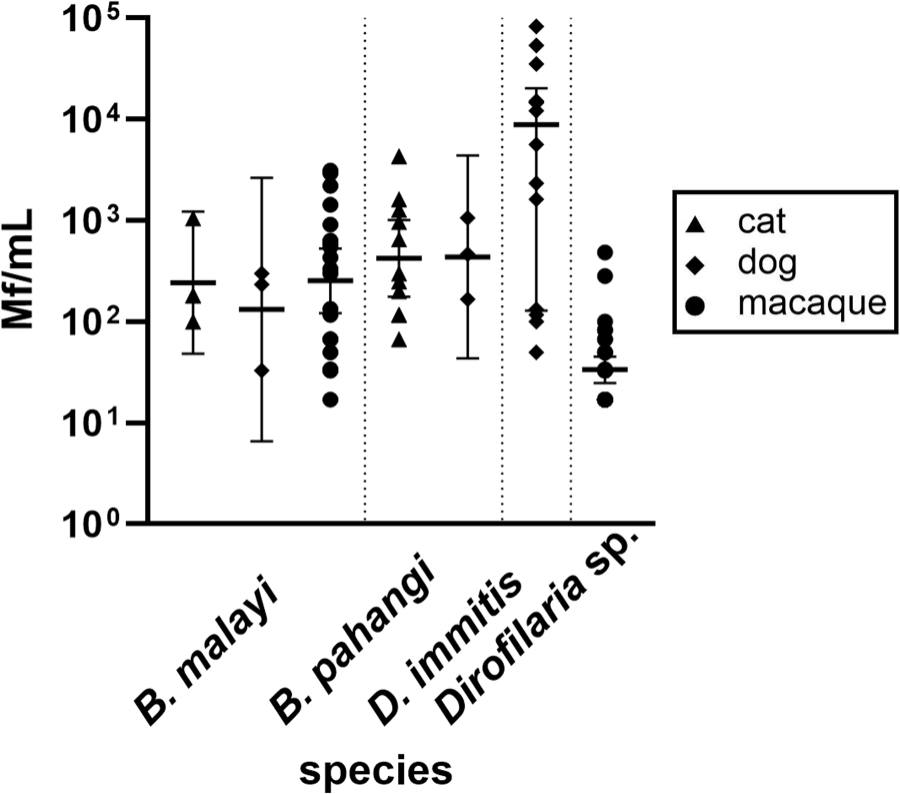
Microfilaria density (logarithmic scale) of different filarial species obtained from different host species. Horizontal bars represent geometric means and error bars are 95% confidence intervals.

Dogs had the lowest geometric mean *B. malayi* Mf density (mean 133 Mf/mL, range 34–233 Mf/mL) followed by cats (mean 244 Mf/mL, range 100–1,050 Mf/mL). Higher average Mf density was detected in macaques (mean 255 Mf/mL, range 17–3,117 Mf/mL) (S1 and S2 Tables). The mean Mf density of *B. pahangi* in cats was 423 Mf/mL (range 67–4,300 Mf/mL) and 437 Mf/mL (range 167-1,067 Mf/mL) in dogs. The mean *D. immitis* Mf density in dogs was 3.170 Mf/mL (range 50–83,330 Mf/mL). The mean Mf density for the unidentified *Dirofilaria* sp. in macaques was 34 Mf/mL (range 17–484 Mf/mL) (Fig 3). One sample from a macaque in Selat Nasik was considered to contain Mf of an unknown species with an exceptionally high Mf density (17,150 Mf/mL, not included in Fig 3). These results show that Mf density varies not only by filarial species but also by host species.

Moreover, the prevalence and density of *B. malayi* Mf varied by village. In Kembiri, infections were only observed in macaques. In Lassar, infections were detected in macaques and in one cat. In Petaling, infections were found in cats and macaques. In Selak Nasik infections were detected in all tested animal species. In Sulat Gual and in Kacang Butor (not endemic for *B. malayi* in humans) none of the tested animals were microfilaremic for *B. malayi* (S1 and S2 Tables).

### Detection and species identification of filarial DNA in animal blood by qPCR

Filarial genomic DNA was detected in 19.6% (97/495) of the animal samples. The *B. malayi*-specific qPCR was positive for 7.1% (35/495) of the animal samples. *B. malayi* DNA was detected in 12 (4.1%) cat samples, one (2.4%) dog sample and 22 (13.5%) macaque samples (S1 and S2 Tables). The *B. pahangi*-specific qPCR detected the parasite DNA in ten (3.4%) and seven (17.1%) samples from cats and dogs, respectively. All macaque samples were negative for *B. pahangi* DNA. *D. immitis* DNA was detected in 17 (39.0%) dog samples but not in blood samples from other animals. 16.6% of the macaque samples were positive only in the pan-filarial qPCR including 19 samples with Mf of the unknown *Dirofilaria* species detected using microscopy and one sample with Mf of an un filarial species. For species identification, single Mf were used for whole genome amplification and qPCR. The population genomic characterization of Mf are the objective of another manuscript. Co- infections were confirmed in three dog samples and one cat sample: one dog sample was positive for three filarial species (*B. malayi/ B. pahangi/ D. immitis*), and dual infections were detected in samples from two dogs (*B. pahangi/ D. immitis*) and one cat (*B. malayi/ B. pahangi*).

### Comparison of morphological and qPCR detection of microfilariae

Data on methodology reliability agreement between morphological identification and qPCR are shown in Table 2. The methodology reliability agreement for detecting any filarial species irrespectively of the species, for detecting *B. malayi* and for detecting *B. pahangi* showed a substantial agreement (Cohen’s κ 0.80, 0.77 and 0.77 respectively), The methodology reliability agreement showed a nearly perfect agreement for *D. immitis*. We found a discordance in 38 samples between microscopic result and qPCR result. This includes results from 11 cats, 11 dogs and 16 macaques. For 13 samples (seven macaque, five dog, and one cat) that were negative by microscopy, none of the targeted species were detected by species-specific qPCR. The disagreement between morphology (Mo) and quantitative PCR (qPCR) results are outlined in detail in S1 and S3 Tables.

**Table 2:** Methodology reliability agreement between morphology (Mo) and quantitative PCR (qPCR).

Comparison	Mo^+^/qPCR^+^	Mo^+^/qPCR^-^	Mo^-^/ qPCR^+^	Mo^-^/qPCR^-^	Cohen’s kappa	95% CI
Filarial nematode	74	6	23	392	0.80	0.73-0.87
*B. malayi*	24	5	11	455	0.77	0.65-0.89
*B. pahangi*	12	2	5	476	0.77	0.60-0.93
*D. immitis*	14	0	3	478	0.90	0.79-1.01

Abbreviations: CI, confidence interval

## Discussion

As expected, this study detected *B. malayi* Mf in cats and dogs. However, the high prevalence and Mf densities for *B. malayi* in macaques were not expected. Microfilariae of *B. pahangi*, *D. immitis* and an unknown *Dirofilaria* species were also found in these animals, and they are potential confounders for species identification by microscopy. We showed that qPCR is very helpful for species-specific detection and confirmation of Mf identification, especially for samples with low Mf densities.

A post-TAS-3 surveillance study in 2021 showed that in Belitung was still endemic for *B. malayi* in humans despite five rounds of effective MDA years earlier [13]. It is possible that the TAS strategy which focuses on antibody surveys of young children failed to detect persistent infections in adults or reemerging infections occur due to transmission from an animal reservoir. There are many reports of natural *B. malayi* infection in cats, dogs and macaques [30]. However, a recent study from other districts of Indonesia (Bengkulu, South-West Sumatra) detected no *B. malayi* Mf in 30 dogs, 40 cats and 30 macaques [31]. Another study sampled 84 cats, 3 dogs and 30 macaques from Belitung and found Mf for *B. malayi* in one macaque [12]. Our study is the first that compared Mf detection and species identification by microscopy with DNA detection of filarial parasites in different host species in Indonesia.

The growth of the human population and increasing agricultural and mining activities puts humans and their companion animals closer to wildlife. This increased proximity increases the risk of pathogen spill over events. Nonhuman primates are of particular concern due to their close relation to humans and shared pathogens [32,33]. One example is the increase of cases of simian malaria in humans [34]. Within primates experimental successful *B. malayi* infection in long tailed macaques (*Macaca irus* synonymous to *M. fascicularis*), rhesus macaques (*M. mulatta*), slow loris (*Nycticebus coucang*), black-crested Sumatran langur (*Presbytis melalophos*) and silvered leaf monkey (*Trachypithecus cristatus*) were reported [35,36]. However, Edeson et al. [35], reported many decades ago that cats appear to be a suitable host for *B. malayi* whereas long-tailed macaques appear to be resistant. In this study we observed a higher prevalence in naturally infected macaques (13.5%) compared to cats (4.5%) based on qPCR. Additionally, we also observed a higher Mf density in macaques (mean 255 Mf/mL) compared to the cats (mean 244 Mf/mL) and dogs (mean 133 Mf/mL). The samples size for dogs (n = 41) is considerably smaller than that for cats (n = 291) and macaques (n = 163). This discrepancy, which is attributable to local religious and cultural practices may impact the accuracy of Mf prevalence estimation for dogs. Nevertheless, the lower abundance of dogs in the study villages indicates a low reservoir capacity for dogs in these areas.

Studies in Thailand treated domestic animals that could act as reservoir for *B. malayi* with anti- filarial drugs [37,38]. While this may help to locally eliminate *B. malayi* infection in areas without free ranging macaques, it is unlikely to have a long-lasting impact in areas with infected macaques. *M. fascicularis* is locally abundant in some areas, such as in Sabah, Malaysia, and their short-term home range can vary between 26 and 45 hectares [39]. Anti-filarial treatment of wild macaques to eliminate zoonotic *B. malayi* is unlikely to be a cost-effective measure to eliminate LF.

It is possible that *B. malayi* transmission within the macaque population is independent of humans, given its high prevalence after extensive MDA to humans. However, infected animals were only found where human infections were also present. Thus, the mere presence of macaques is not sufficient for sustained transmission of *B. malayi* in Belitung. We think it is likely that there is bidirectional transmission of *B. malayi* between humans and animals in this area, but longitudinal data are needed to elucidate reservoir dynamics.

Macaques were trapped at the edges of large palm oil plantations close to human settlements. Water reservoirs of the plantation and the large, vegetation-rich plantation draining systems are perfect breeding sites for many species of mosquitos. Ecological factors, such as humidity and temperature can affect parasite development and vector survival [40]. It is not known which local mosquito species is the main vector for *B. malayi* transmission between humans and animals. To address this question, in an ongoing study, we collected 82,000 mosquitoes in the endemic areas, mainly species of the genera *Amigeres*, *Mansonia*, *Aedes* and *Culex* and examined most of them using pool-screen qPCR. The abundance of competent mosquito vectors and the density of definitive hosts may determine the sustainability of the transmission cycle [41–43].

The detected species composition of Mf and their density likely depend on the blood collection time because filarial species may show different periodicities of Mf [7]. The collection time may potentially lead to an underestimation of infections of other filarial species due to the variation in their Mf periodicity. Furthermore, the composition of filarial species detected varied by host species. *B. malayi* infections were found in all examined animal species. No infections with *B. pahangi* or *D. immitis* were detected in macaques. Experimental studies suggested the potential infection risk with *B. pahangi* in humans and macaques [44,45], but primates seem less susceptible in contrast to carnivores. Case reports mention that natural infections with *B. pahangi* in humans are rare [14,46].

Sensitivity of morphological and molecular detection of Mf depends on the volume of examined blood. The volume used for microscopy and DNA isolation differed by 10 μL of blood (60 vs 50 uL). Despite of the higher volume used for microscopy, 23 samples were negative by microscopy but positive by qPCR. In contrast, only six samples were positive by microscopy but negative by qPCR. Thirteen samples were positive by pan-28S qPCR, but negative by microscopy and the other molecular assays. We were not able to sequence PCR products from these samples. It is possible that these animals had infections with low blood Mf counts that were not detectable by microscopy, or they may have had amicrofilaremic infections [47]. This shows the superior sensitivity of the qPCR compared to microscopy.

The comparison of Mf detection by microscopy and by qPCR showed that more samples were Mf positive by qPCR and that Mf specification based on morphological characters is not always accurate. The discrepancy and challenges between morphological and molecular identification and detection has also been demonstrated for other parasite species [48–50]. Statistical analyses based on Cohen’s Kappa takes into account the higher number of negative samples, which can influence the agreement. More positive results would be needed to be able to make a better statement about the methodology reliability agreement. The nearly perfect agreement for *D. immitis* based on Cohens Kappa can be explained by the distinct morphological features, the larger size and the high density of *D. immitis* Mf in dogs compared to *Brugia* spp. In contrast, the morphological species identification of *Brugia* Mf is more difficult. Cohen’s Kappa results show a substantial agreement between the morphological and molecular identification of *B. malayi* and *B. pahangi.* Based on the morphometric results differences could be confirmed between both species. For example, the ratio of width and cephalic free space is often used as an identification method [51]. In this study for *B. malayi* and *B. pahangi,* the ratio did not show a significant difference, which can be challenging for identification. In addition, *B. pahangi* Mf obtained from different host species showed differences in size. In this study, discordant results between microscopy and molecular analysis were observed for *B. malayi* in 16 samples and for *B. pahangi* in seven samples. Focusing on reliable detection of *B. malayi* in animals, misidentification or missing detection due multiple species infection can only be ruled out by a combination of microscopy and molecular methods. For *W. bancrofti*, morphometric variation was explained due to its wide geographical distribution [52], but morphometric studies of zoophilic *B. malayi* from the same endemic area in different host species are lacking. Since we did not observe any difference in size between Mf from a cat and macaques compared to humans, the size difference could be also due to natural variations in different hosts, different nutrition or even host species adaption. It has to be noted that staining and measurement techniques were identical for all Mf and a bias can be excluded. Although microscopy is more affordable and practical for diagnosis in field study sites the number of false-negative samples by microscopy including eight *B. malayi* infections should highlight the importance of qPCR as diagnostic tool. In addition to covering the spectrum of possible further co-infection with other species, to detect novel species and to understand the parasite population in humans and animals, further molecular methods such as high throughput sequencing are required [53].

While it appears plausible that *B. malayi* parasites found in humans and in animals in the same area could belong to the same population, it is possible that they have separate transmission cycles, with different primary vector species as described for other vector-borne, zoonotic pathogens [54]. Population genomic studies of the parasite are needed to decipher the exact role of animals as reservoirs that contribute to human *B. malayi* infection. This type of information will be crucial for developing strategies to interrupt *B. malayi* transmission in areas like Belitung. Enhanced surveillance including special surveys of humans and xenomonitoring, and intervention such as MDA or targeted treatment of high-risk groups may be necessary to eliminate brugian filariasis in humans in endemic areas with zoophilic strains. Further research is needed to provide vector and molecular data to fully understand the epidemiology and potential transmission of *B. malayi* from macaques to humans which may lead to strategies for preventing zoonotic transmission.

## Conclusion

*B. malayi* infection was detected in cats, dogs and macaques collected in or near villages with infected humans. Macaques (with the highest Mf prevalence and density) appear to be the most important reservoir for the infection in animals. Ecological changes may have led to increased proximity between free ranging macaque groups and human settlements. Unlike humans in the area, animals were commonly infected with additional filarial species. We also found that microscopical detection and identification of Mf was not always accurate. Therefore, qPCR should be used to confirm microscopy results when this is feasible.

## Supporting information

S1 Table Metadata, microscopy, qPCR and agreement results between morphology (Mo) and quantitative PCR (qPCR) per individual animal. (XLSX)

S2 Table Total number of collected and infected animals with Brugia malayi, prevalence based on morphology and real-time PCR and Mf density within different villages. (XLSX)

S3 Table Disagreement between morphology (Mo) and quantitative PCR (qPCR) per individual host species. (XLSX)

S4 Table Real-time qPCR primers/probes and reaction conditions. (XLSX)
